# Supplementary material for: The choice-wide behavioral association study: data-driven identification of interpretable behavioral components
Source: Nat Commun. 2026 Aug 13;17:9746. doi: 10.1038/s41467-026-76681-3 (PMC13572513; doi:10.1038/s41467-026-76681-3)
Supplement: Supplementary file 1 [file 41467_2026_76681_MOESM1_ESM.pdf]

Supplementary Figure 1

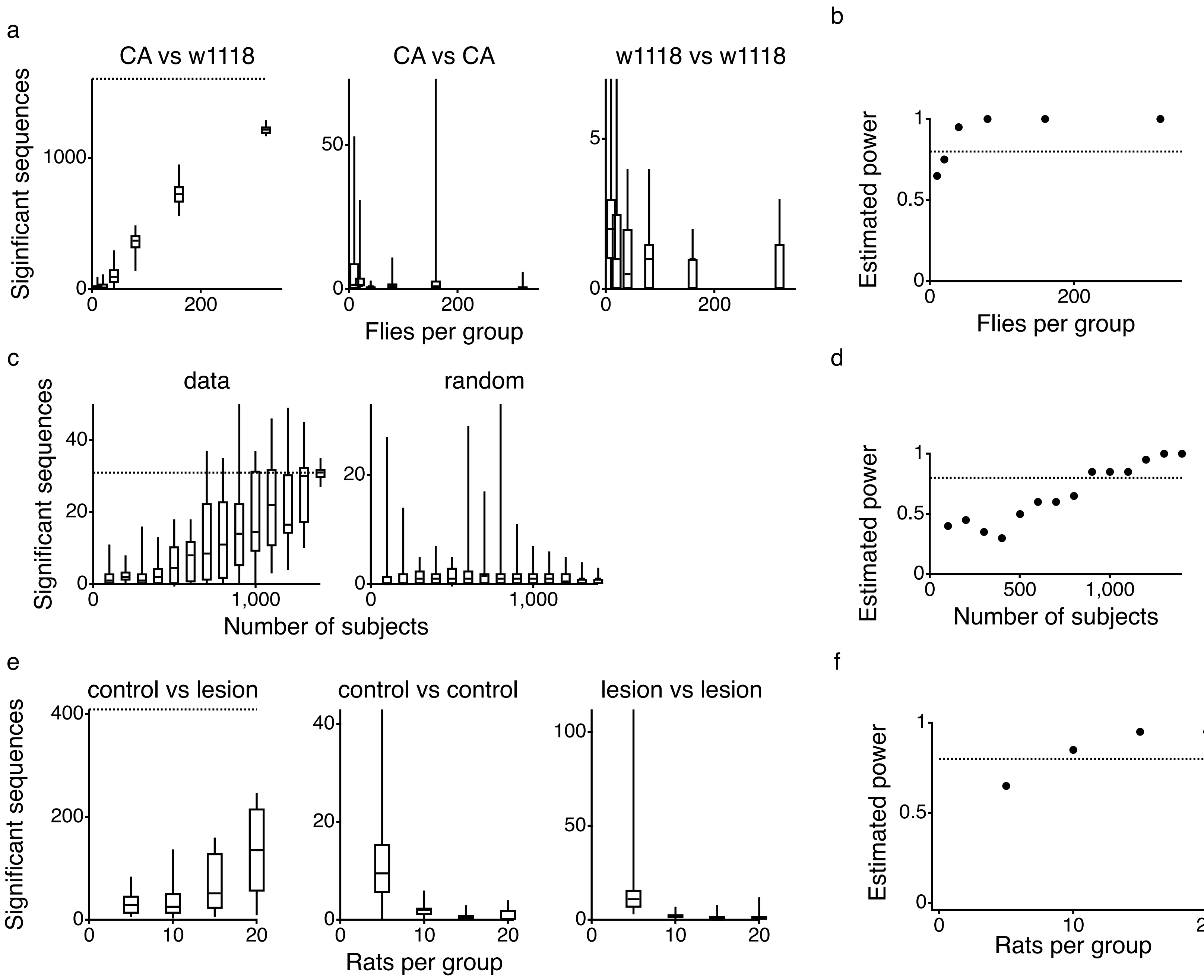

Supplementary Figure 1. Sample size calculations for CBAS. Power analyses for CBAS using random subsamples of the fly, human, and rat datasets. (**a**) Fly data. Left: number of significant sequences identified by CBAS in random subsamples of the two fly strains. Middle and right: numbers of significant sequences obtained when comparing nonoverlapping subsamples from the same CA or w1118 strain. (**b**) Estimated statistical power as a function of sample size for the fly dataset. (**c**) Human data. Left: number of significant sequences correlated with CBIT in random subsamples. Right: number of significant sequences obtained using randomly generated CBIT scores drawn from the same distribution as the observed data. (**d**) Estimated statistical power for the human dataset. (**e**) Rat data. Left: number of significant sequences identified in random subsamples of the control and hippocampal-lesioned groups. Middle and right: numbers of significant sequences obtained by comparing nonoverlapping subsamples within the control or lesion groups. (**f**) Estimated statistical power for the rat dataset. In **a**, **c**, and **e**, box plots show the median, interquartile range, and full range across subsamples. Horizontal dotted lines indicate the number of significant sequences identified in the complete dataset. In **b**, **d**, and **f**, the horizontal dotted line indicates 80% power.

Supplementary Figure 2

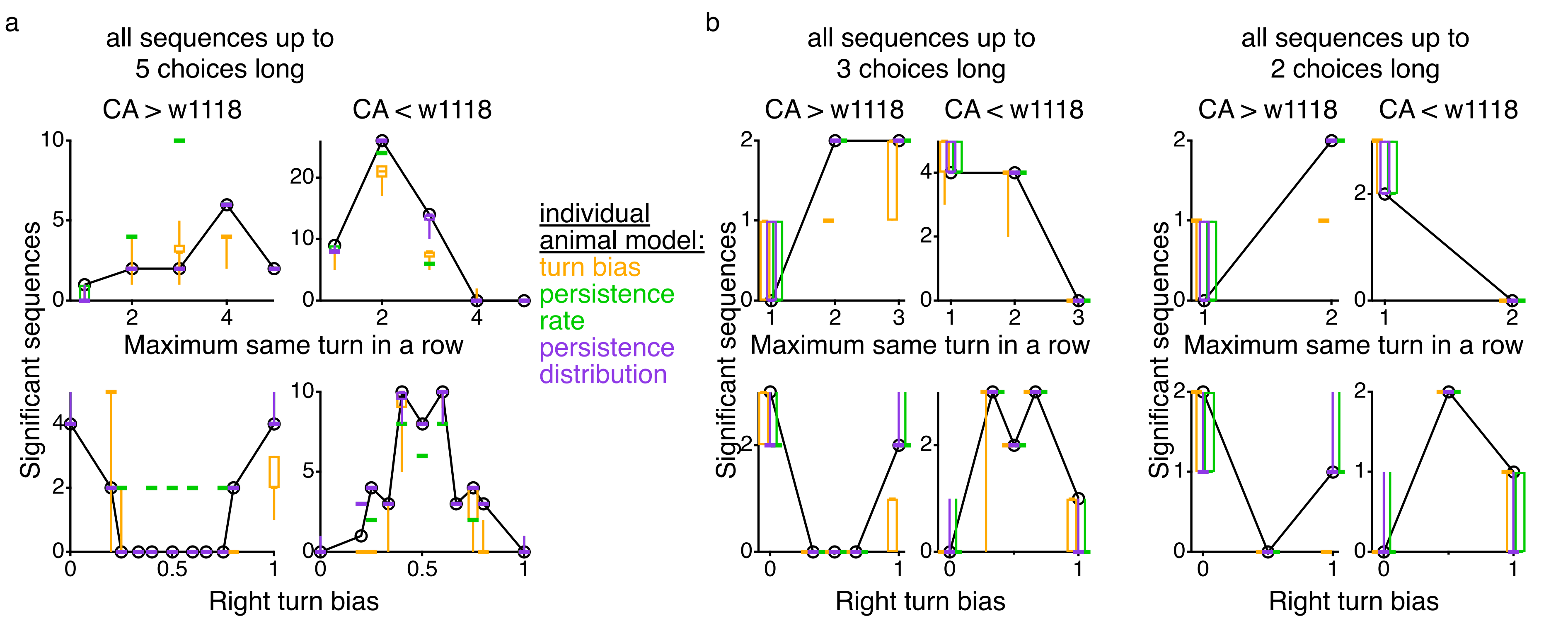

Supplementary Figure 2. CBAS reaches similar conclusions across different maximum sequence lengths. (a) CBAS applied to the fly spontaneous alternation dataset using sequences up to length 5. Numbers of significant sequences identified for the data (black) and three generative models of fly behavior (colored box plots): turn bias, persistence rate, and persistence distribution. Top panels group sequences by persistence (maximum number of consecutive turns in the same direction), and bottom panels by the fraction of right turns. Left and right columns quantify sequences occurring more frequently in the CA and w1118 strains, respectively. Model results are based on 100 simulated datasets. Boxes indicate the median and interquartile range; whiskers indicate the full range. **(b)** Same analysis using maximum sequence lengths of 3 (left) and 2 (right). Although shorter sequences distinguish the turn bias model from the data, only longer sequence lengths discriminate between the persistence rate and persistence distribution models.

	control rat sequence usage rate	lesion rat sequence usage rate	sufficient power	significance
**32**12**3**1	0.04	0	no	
**32**12**3**2	1.67	0.85	yes	control > lesion
**32**12**3**4	0	0.05	no	
**32**12**34**	7.11	3.00	yes	control > lesion
**32**12**3**5	0.11	0	no	
**32**12**3**6	0.07	0.03	no	
→ **32**12**3**	9.00	3.92	yes	control > lesion
1**32**12**3**	0	0	no	
2**32**12**3**	0	0	no	
4**32**12**3**	3.61	1.56	yes	control > lesion
**432**12**3**	3.57	1.64	yes	control > lesion
5**32**12**3**	0.80	0.15	yes	control > lesion
6**32**12**3**	0.15	0.10	no	

REWARDED
unrewarded

Supplementary Figure 3. Definition of "complete" sequences. Example illustrating the identification of a complete sequence. The target sequence (32123; arrow) is compared with all longer sequences containing that subsequence. Bold numbers indicate rewarded choices. For each sequence, the usage rate in control rats, whether the sequence exceeds the minimum usage required for 80% statistical power, and whether CBAS identifies it as significantly different between control and hippocampal-lesioned rats are shown. A sequence is defined as *complete* if all sufficiently frequent longer sequences containing it differ significantly between groups in the same direction. In this example, all adequately powered extensions of the target sequence occur more frequently in control than hippocampal-lesioned rats, satisfying the criterion for a complete sequence.

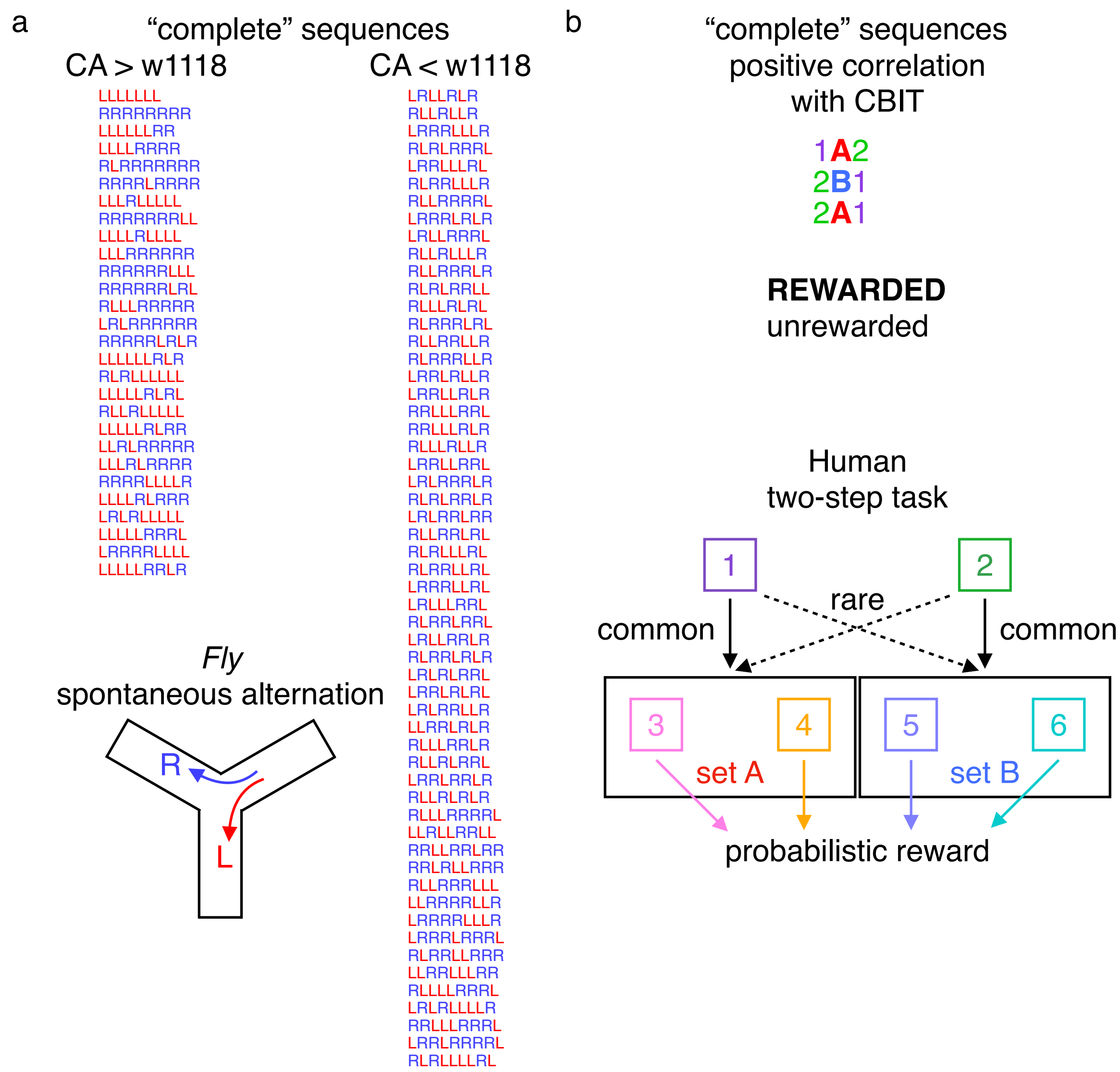

Supplementary Figure 4. "Complete" sequences for the fly and human tasks. (a) Complete sequences identified by CBAS for the fly spontaneous alternation task. **(b)** Complete sequences identified by CBAS for the human two-step task. No complete sequences were negatively correlated with CBIT. Uppercase bold letters indicate rewarded choices.

Supplementary Figure 5

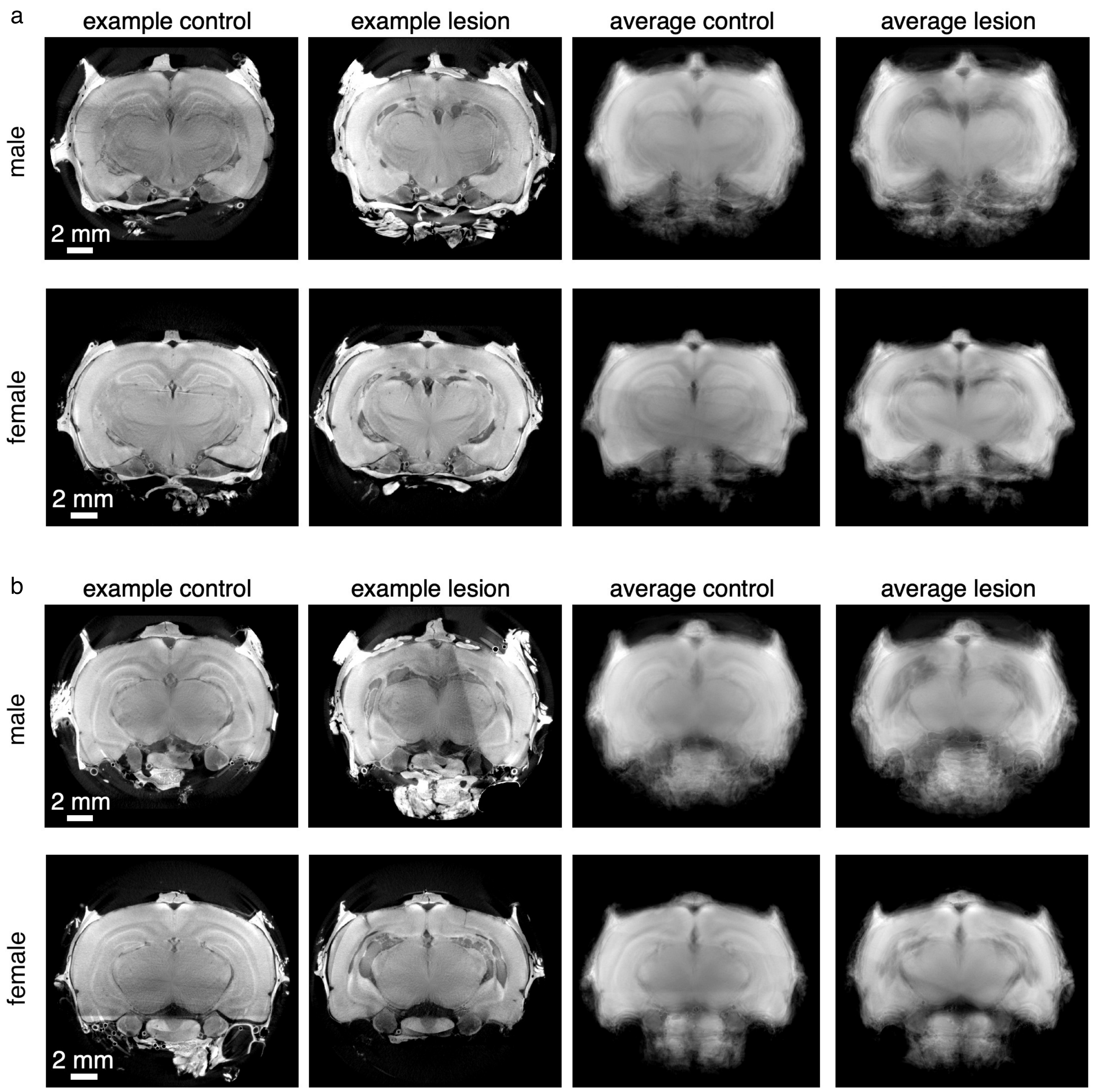

Supplementary Figure 5. Micro-CT confirmation of hippocampal lesions. Representative virtual coronal sections through anterior (**a**) and posterior (**b**) hippocampus from male and female control and hippocampal-lesioned rats. Corresponding average images are shown for each group following linear registration. Hippocampal cell layers remain intact in control brains but are largely absent in lesioned brains, confirming successful targeting of the hippocampus. Averaging introduces some blurring because of imperfect registration across animals; however, the hippocampal cell layers remain clearly visible in the control averages and absent in the lesion averages. Example control and lesion images are shown on the same intensity scale, whereas the averaged images are displayed with increased gain to better visualize anatomical features.
