# Supplementary material for: The choice-wide behavioral association study: data-driven identification of interpretable behavioral components
Source: Nat Commun. 2026 Aug 13;17:9746. doi: 10.1038/s41467-026-76681-3 (PMC13572513; doi:10.1038/s41467-026-76681-3)
Supplement: Supplementary file 2 [file 41467_2026_76681_MOESM2_ESM.pdf]

nature portfolio

Peer Review File

The choice-wide behavioral association study: data-driven identification of interpretable behavioral components

Corresponding Author: Dr David Kastner

This file contains all reviewer reports in order by version, followed by all author rebuttals in order by version.

Version 0:

Reviewer comments:

Reviewer #1

(Remarks to the Author)
This manuscript describes a new and exciting approach to tackling the complexities of behavioural data across species by using a data-driven analysis method which can identify interpretable behavioural components that are grounded in sequence of choices made by subjects. A very important outcome of this study is that the application of CBAS uncovered a much deeper interpretation of previously characterised data sets so that the core underlying behaviour could be revealed (e.g. flies were persisting in the same direction for a range of turns rather than showing handedness.). I only have a few points for query:

Results
Fig 3a and b: the x axis should include more intervals at 50 and 100.
Fig 3a: Does a ratio of 1 mean the sample size produced the same number of significant sequences as the full data set?
Figure 3b showing a small fraction of sequences found in the smaller data sets but not in the larger data sets is interpreted as false positives. What would cause these false positives and why do they not change by sample size?
Figure 3c was difficult to interpret initially – could the label of the y axis be a bit more explanatory, e.g. shared sequence / different sequences between groups

Figure 4b. The grey bars were difficult to distinguish from the black – suggest and alternate colour.
In the results section for 4b can you please provide an example of a virtual twin choice?

Figure 4c. This figure was also difficult to interpret. Can you provide in the results section a clear summarising sentence here? Does the data suggest that when the subject is using model-inconsistent choices they are unlikely to be using model-based parameters to guide their choice?
I would have liked to have seen a deeper interpretation from the authors in the discussion surrounding the model-inconsistent choices

Figure 5c. Most rats have a Transition normalisation directional inertia of around 0.25. What sequence does this represent? Are you able to provide a key with the inertia score given for each sequence?

A major strength of the study is that it shows how CBAS can be applied across multiple species and tasks. However, I wonder how it could be applied to compare outputs across species performing analogous tasks. For examples, the rat test is very hippocampal dependent and relies on spatial navigation, while the human test is more of a reward-based learning task. It would be interesting to know how CBAS compared across species in more similar spatial navigation learning tasks across species. Could CBAS be harnessed as a tool to aid cross species analysis?

Can the authors further describe the literature surrounding evidence that the hippocampus is responsible for implementing a structuring of task space? Is there any evidence from human or non-human primate models that this is the case?

In the discussion the authors mention that CBAS is not able to just evaluate one group. However, if a set of unique outcomes are uncovered by CBAS can regrouping be applied to the data set based on these outcomes? For example, could all rats that show a higher proportion of a specific sequence of choices then be grouped together irrespective of lesion or non-lesion?

(Remarks on code availability)

Reviewer #2

(Remarks to the Author)
This study proposed to apply one of the established correction methods of multiple statistical comparison, Romano-Wolf correction, to extract significant differences in behavioral choice sequences. This approach is important to assure some conclusions derived by hypothesis-driven approaches. So far, however, the study has not yet sufficiently shown any potential to extract novel biological findings. In addition, I wonder if the proposed approach is truly data-driven. The methodology was previously established, and hence, no novelty exists in the method itself. Therefore, it does not provide sufficient impact to general readers, at least in the present form.

The detail of comments was described in a enclosed review attachment PDF file.

(Remarks on code availability)

Reviewer #3

(Remarks to the Author)
This study introduces the Choice-Wide Behavioral Association Study (CBAS), a novel data-driven method for systematically identifying behavioral components from sequences of discrete choices. Inspired by the logic of genome-wide association studies (GWAS), CBAS evaluates the occurrence of all discrete choice sequences up to a user-defined length and uses a resampling-based multiple comparisons correction (using Romano-Wolf method) to identify sequences that either significantly differ between groups or correlate with a covariate of interest. The authors apply CBAS to three example tasks across three species, namely spontaneous alternation in flies, the two-step task in humans, and spatial alternation in rats, demonstrating the breadth of the method. In each case, CBAS identifies interpretable behavioral differences that are either inconsistent with or go beyond the original conclusions of prior model-dependent analyses and the work that the data came from. For instance, for the rat spatial alternation task, CBAS reveals that the hippocampus supports a specific exploratory strategy characterized by directional inertia and short-range arm transitions, reframing the hippocampal contribution away from simple memory allocation.

The paper addresses an important gap in behavioral neuroscience methodology, and the three diverse applications make a compelling case for the generality of the approach. The statistical framework is rigorous, and the results are clearly presented. Our comments primarily focus on clarifying key methodological choices and strengthening the interpretation of the results.

Major Comments:

1. The GWAS analogy would benefit from a more explicit treatment of the conceptual equivalences (e.g. line 85). The authors motivate CBAS by drawing on GWAS, WES, and WGS, which is a compelling framing. However, the analogy is stated at a high level without spelling out the specific parallels. For instance, it would help the reader to understand how a choice sequence maps onto a base pair or a genetic variant in more detail, what the behavioral equivalent of a phenotype is in this framework, and how the resampling-based correction serves the same role as genome-wide significance thresholds. Making these correspondences explicit would strengthen the conceptual contribution of the paper and help readers from different backgrounds appreciate the logic of the approach.

2. The inability of CBAS to operate on a single group without a covariate is a notable limitation that warrants further discussion. The authors briefly acknowledge in the Discussion that CBAS currently requires either a group comparison or a covariate of interest, and cannot evaluate a single group in an unsupervised manner. However, this seems like a meaningful constraint on the method’s applicability, particularly in exploratory settings where no clear comparison group or covariate exists. It would be helpful if the authors expanded on this limitation, discussed whether there are principled extensions that could address it, and clarified whether existing unsupervised approaches (e.g., clustering-based methods) could be combined with CBAS in such cases.

3. The generalizability of CBAS beyond discrete choices is not fully addressed. CBAS is built around the discretization of behavior into choices, which works naturally for the three tasks presented. However, many behavioral paradigms do not yield such clean discrete representations and many research approaches are using more continuous paradigms. The authors briefly note this limitation but do not discuss what strategies might be used to extend CBAS to richer or higher-dimensional behavioral spaces, for instance through unsupervised discretization of continuous trajectories. A more thorough exploration of this boundary condition would help the reader understand where CBAS is and is not applicable.

4. The sensitivity of the conclusions to the choice of maximum sequence length is not assessed. The maximum sequence length is a key user-defined parameter in CBAS, set to 10 for flies, 4 for humans, and 6 for rats, but the rationale for these specific choices is not clearly explained, and no sensitivity analysis is provided. It is not obvious that the key conclusions would hold under different choices of this sequence length parameter. For example, would the same interpretable patterns in

the fly data emerge if sequences up to length 6 or 8 were used instead of 10? The authors should either provide a justification for these choices or demonstrate that the results are robust to reasonable variation in this parameter.

5. The distinction between 'persistence rate' and 'persistence distribution' is not clearly explained (e.g. line 156). The authors compare three generative models of fly behavior, namely turn bias, persistence rate, and persistence distribution, and show that only the persistence distribution model matches the CBAS output. However, the difference between persistence rate and persistence distribution is not made sufficiently clear. Specifically, it is not obvious what additional information the distribution captures beyond the rate, and why this distinction matters for generating realistic sequences and why this distinction is functionally useful.

6. It is unclear how the replication dataset addresses the circularity concern (as in Figure 6). The authors use the replication dataset to validate the hypothesis of reduced directional inertia in hippocampal lesion rats. However, the replication uses the same task, the same apparatus, and the same general experimental design as the initial dataset, and is simply a smaller independent cohort. Given that the hypothesis was derived directly from the CBAS output on the initial dataset, and that the same metric (transition normalized directional inertia) is applied in the replication, it is not clear that this fully resolves the circularity concern the authors themselves raise. Could the authors clarify what specific aspect of circularity the replication is intended to address, and what would constitute a stronger test?

7. The notion of 'model-inconsistent' choices needs to be better grounded in decision theory. The authors identify sequences of the form 1A2 and 2B1, where a subject is rewarded on the common side but then selects the image that will rarely return them there, as 'model-inconsistent.' While this is an interesting empirical observation, the conceptual status of these choices is not clearly defined. They are inconsistent with both model-based and model-free learning, but what alternative decision process would produce them? The authors suggest these choices may be related to CBIT without explaining the mechanism. This example may provide a test case how to interpret the CBAS-based discovery for functional implication with respect to disorders or other mechanisms in the brain. A more careful discussion of what model-inconsistent choices reflect, whether they constitute a distinct behavioral mode, a failure of attention, or something else entirely, would substantially strengthen the interpretation of the two-step task results.

Minor Comments:

1. Figure 1b: The rate values reported (e.g., 9.9 per animal for the CA strain) lack a unit. It would be helpful to clarify whether this is sequences per 250 choices, per session, or some other normalization.

2. Line 795: typo: "odel" should be "model."

3. Line 362: The section header reads "DISCUSSSION" and should be corrected to "DISCUSSION."

(Remarks on code availability)

Reviewer #4

(Remarks to the Author)
I co-reviewed this manuscript with one of the reviewers who provided the listed reports. This is part of the Nature Communications initiative to facilitate training in peer review and to provide appropriate recognition for Early Career Researchers who co-review manuscripts.

(Remarks on code availability)

Version 1:

Reviewer comments:

Reviewer #1

(Remarks to the Author)
The authors have addressed all comments raised in a comprehensive manner. I believe this manuscript is ow suitable for publication.

(Remarks on code availability)

Reviewer #2

(Remarks to the Author)
My point is not whether novel findings exist in this study. My concern is that the findings provided here could be derived even in standard hypothesis-driven approaches. Hence, I cannot say that this work has sufficiently shown the potential of CBAS.

The authors provided only the rebutal to my concern: “Whether the results could have been identified in other ways is not irrelevant to the present study”. If so, the authors should withdraw their claim as if CBAS provided information unexpected in standard hypothesis-driven approaches. They should clarify as a limitation that their findings could be derived in standard hypothesis-driven approaches.

Revision proposals:

L23: “showing that it provides unexpected and interpretable information in each case.“
-> “showing that it provides interpretable information in each case.“

L407: “We have presented a general data-driven behavioral analysis method, CBAS,”
-> “We have presented a behavioral analysis method, CBAS, towards the data-driven end of the spectrum from the top-down/model-
dependent one”

L420-422: “Somewhat surprisingly, in all tasks, CBAS provides information and generates interpretations that are either inconsistent with, or unexpected from, the prior understanding of these data and tasks.”
-> “Somewhat surprisingly, in all tasks, CBAS provides information and generates interpretations that are either inconsistent with, or unexpected from, the prior understanding of these data and tasks. Although interpretable information could be derived by top-down/hypothesis-driven approaches, CBAS provides a more unbiased consequence than top-down biases.”

(Remarks on code availability)

Reviewer #3

(Remarks to the Author)
The authors have addressed all of our original comments. Although they could have done more to quantify the sensitivity of the conclusions to the choice of maximum sequence length, it is acknowledged that they (1) provided the reasoning for the sequence length for each dataset, (2) provided an impression to the readers that the choice of sequence length will likely depend on the specific task context/dimensions, and (3) tested different lengths for the fly dataset to provide evidence on the flexibility and also the limitation of choosing different sequence lengths. We hope the approach from this manuscript will be impactful in the field of complex behavioral analyses.

(Remarks on code availability)

Reviewer #4

(Remarks to the Author)
I co-reviewed this manuscript with one of the reviewers who provided the listed reports. This is part of the Nature Communications initiative to facilitate training in peer review and to provide appropriate recognition for Early Career Researchers who co-review manuscripts.

(Remarks on code availability)

Open Access This Peer Review File is licensed under a Creative Commons Attribution 4.0 International License, which permits use, sharing, adaptation, distribution and reproduction in any medium or format, as long as you give appropriate credit to the original author(s) and the source, provide a link to the Creative Commons license, and indicate if changes were made.
In cases where reviewers are anonymous, credit should be given to 'Anonymous Referee' and the source.
The images or other third party material in this Peer Review File are included in the article's Creative Commons license, unless indicated otherwise in a credit line to the material. If material is not included in the article's Creative Commons license and your intended use is not permitted by statutory regulation or exceeds the permitted use, you will need to obtain permission directly from the copyright holder.
To view a copy of this license, visit https://creativecommons.org/licenses/by/4.0/

REVIEWER COMMENTS

Reviewer #1 (Remarks to the Author):

This manuscript describes a new and exciting approach to tackling the complexities of behavioral data across species by using a data-driven analysis method which can identify interpretable behavioral components that are grounded in sequence of choices made by subjects. A very important outcome of this study is that the application of CBAS uncovered a much deeper interpretation of previously characterized data sets so that the core underlying behavior could be revealed (e.g. flies were persisting in the same direction for a range of turns rather than showing handedness.). I only have a few points for query:

Thank you very much for this very positive assessment of our paper and for your helpful comments.

Results

Fig 3a and b: the x axis should include more intervals at 50 and 100.

For Fig 3 a-c, we have included extra x-axis tics demarcating every unit of 50 and labels for every unit of 100.

Fig 3a: Does a ratio of 1 mean the sample size produced the same number of significant sequences as the full data set?

We have added text in the figure legend to clarify this. "A value of 1 means that the CBAS on the subsampled population identified all of the sequences identified in the full population."

Figure 3b showing a small fraction of sequences found in the smaller data sets but not in the larger data sets is interpreted as false positives. What would cause these false positives and why do they not change by sample size?

As we are controlling the false discovery proportion and not the familywise error rate, we expect some level of false positives. The absence of substantial change across sample sizes is a good illustration of the control provided by the Romano Wolf method. We thank the reviewer for pointing this out and have added text to the description of this figure in the manuscript: "We next evaluate the fraction of the sequences identified by CBAS for the smaller sized groups that are not identified in the CBAS on the full dataset (which we might interpret as false positives). Since we control the median false discovery proportion at 5% and not the familywise error rate, we do not expect this value to be zero; however, it was consistently small across all the group sizes evaluated (Fig 3b). The medians across the different repeats of the same sample sizes across the different sample sizes are all less than 2%, and the values do not actually get grossly larger with the smaller sample sizes. This is a testament to, and a good illustration of, the control provided by the Romano-Wolf method for multiple comparisons correction."

Figure 3c was difficult to interpret initially—could the label of the y axis be a bit more explanatory, e.g. shared sequence / different sequences between groups

Thank you for this suggestion. We apologize that the previous label was insufficiently informative as to what was being displayed. We have updated the label to be "Shared sequences across CBAS runs," to better highlight that the plot is comparing CBAS runs on different groups of animals to compare the similarity of the output.

Figure 4b. The grey bars were difficult to distinguish from the black—suggest an alternate color.
In the results section for 4b can you please provide an example of a virtual twin choice?

Thank you for these suggestions. We have changed the color of the grey bars in Figure 4b and have added clarifying text to explain what we mean by virtual twin choices (this is a term in increasing used in

the modeling community—we now explain it: "For the human data, the virtual twin for each subject had choices generated by the model using the best fit parameters for that subject."

Figure 4c. This figure was also difficult to interpret. Can you provide in the results section a clear summarizing sentence here? Does the data suggest that when the subject is using model-inconsistent choices they are unlikely to be using model-based parameters to guide their choice?
I would have liked to have seen a deeper interpretation from the authors in the discussion surrounding the model-inconsistent choices

Apologies for this. In order to summarize the implication of the finding being described in fig 4c better, we added the text: "To understand this, take the case that the CBIT values for the subjects were positively correlated with their rate of making model inconsistent choices, but did not differ at all in their model-based planning. Then, if we fit those subjects with the MB/MF RL model and used the hypothesis-driven analysis typically applied to the two-step task to interpret the results, we would see what amounts to a spurious negative correlation between model-based planning and CBIT."

Furthermore, we added more description in the discussion section adding extra interpretation of the implications of model inconsistent choices and their potential relationship to CBIT. "Possible ways in which model-inconsistent choices positively correlate with CBIT include an increase in exploration in the context of the lower task success that has been shown to associate with OCD[53]. That lower task success could lead to failures in both model-based and model-free decision making, which then manifest as more model inconsistent choices. Another route would be an increase in a desire for symmetry that can be a prominent component of OCD[54]. That desire for symmetry could lead to an evening out of choices to both of the first images independent of the potential for success of those choices. These would then naturally be model inconsistent."

Figure 5c. Most rats have a Transition normalization directional inertia of around 0.25. What sequence does this represent? Are you able to provide a key with the inertia score given for each sequence?

To describe figure 5c better, we added the text: "When an animal performs the task perfectly, making choices in the sequence **3234323** etc., they will have a transition normalized directional inertia value that averages to 0.5. The control rats reach an average value of ~0.4 at choice 800, and the lesion animals reach a value of ~0.38 at choice 800."

A major strength of the study is that it shows how CBAS can be applied across multiple species and tasks. However, I wonder how it could be applied to compare outputs across species performing analogous tasks. For examples, the rat test is very hippocampal dependent and relies on spatial navigation, while the human test is more of a reward-based learning task. It would be interesting to know how CBAS compared across species in more similar spatial navigation learning tasks across species. Could CBAS be harnessed as a tool to aid cross species analysis?

This a very interesting point and could be an important use-case for CBAS. We added text in the discussion section to highlight this. "In this work when comparing groups, we focused on groups of animals within the same species; however, CBAS could just as easily compare the same behavior across different species[64], enabling data-driven information for cross species insights."

Can the authors further describe the literature surrounding evidence that the hippocampus is responsible for implementing a structuring of task space? Is there any evidence from human or non-human primate models that this is the case?

In the discussion, we now cite recent experimental results from both mice and humans concerning the role of the hippocampus, along with literature on modeling. "This strategy is far more consistent with the hippocampus implementing a structuring of the task space[56] as described in recent models of

hippocampal function[57,58] and reported in recent experimental results across species[59–61], than with the typical memory function assumed in the prior studies of spatial alternation behavior."

In the discussion the authors mention that CBAS is not able to just evaluate one group. However, if a set of unique outcomes are uncovered by CBAS can regrouping be applied to the data set based on these outcomes? For example, could all rats that show a higher proportion of a specific sequence of choices then be grouped together irrespective of lesion or non-lesion?

We have added further text to the discussion section to describe possibilities for surmounting this limitation: "If there is only a single group of subjects with no experimentally defined covariate, but a regular clustering algorithm detects structure, then CBAS can be run on the output of that. In future work, we will examine an unsupervised application of CBAS in which robust cluster structures arise from random initial conditions."

Reviewer #2 (Remarks to the Author):
This study proposed to apply one of the established correction methods of multiple statistical comparison, Romano-Wolf correction, to extract significant differences in behavioral choice sequences. This approach is important to assure some conclusions derived by hypothesis-driven approaches. So far, however, the study has not yet sufficiently shown any potential to extract novel biological findings. In addition, I wonder if the proposed approach is truly data-driven. The methodology was previously established, and hence, no novelty exists in the method itself. Therefore, it does not provide sufficient impact to general readers, at least in the present form.

Thank you for this comment. The novelty of CBAS is not due to the use of the Romano-Wolf method. CBAS is innovative in its conceptualization that behavior can be broken down into all sequences of discrete units (up to a user-defined length) and that this can form the basis for finding statistically reliable differences between groups. We are not aware of any other approach in the literature that attempts anything like this. We were puzzled by the comment that CBAS does not extract novel biological findings: as we describe in the manuscript (and is kindly acknowledged by this reviewer), CBAS applied to all three behavioral paradigms produces novel understanding of the behaviors. Whether the results could have been identified in other ways is not irrelevant to the present study. The fact is that these insights were not previously described for these data and behavioral paradigms. Furthermore, the same basic CBAS methodology was able to extract out the relevant information for all three of the behaviors; highlighting the generality and usefulness of CBAS.

Of course, the reviewer is correct that we did not invent any new statistical technique for testing statistical reliability. We did this through the application of the Romano-Wolf resampling based multiple comparisons-correction method. Furthermore, for that statistical control it incorporates other methods developed by Romano and colleagues into the resampling multiple comparisons correction framework to enable false discovery proportion control and correction across correlation values.

In sum, the novelty is in the interdisciplinary combination of disparate perspectives and approaches and the substantial enhancement in our ability to interpret behavior.

1. Superiority of CBAS to previous hypothesis-driven approaches is unclear.
In the case of the spontaneous alternation, behavioral sequences are simple binary ones. Therefore, basically possible and interpretable hypotheses are restricted. The revealed differences can be also derived by hypothesis-driven approach focusing on temporal correlations of stay or switch sequences. In the case of the two-step task, the difference of model-inconsistent choices is interesting. But this is contained in the standard stay probability analysis. It is not a finding that became possible because of CBAS. In the case of the spatial alternation, hypotheses about directional features are easily expected. This case is also not a finding that became possible because of CBAS. Although this study has not

demonstrated here, even if CBAS could extract unexpected complicate sequences, I wonder if we can interpret the feature of the sequences.

The reviewer's point about the difficulty of interpreting the output of CBAS is a very important one. We introduced the notion of "complete" sequences exactly for that purpose. We have every expectation that there is future work to be done in developing further follow up analyses to interpret the output of CBAS and point to that goal in the discussion section.

Additionally, CBAS provides a different perspective for analyzing behavioral data. By richly comparing the groups or evaluating correlations with a covariate of interest, CBAS provides a rigorous framework upon which an interpretation can be built. This has been absolutely critical for the field of Genomics and can be viewed as rather lacking in many behavioral studies.

So far, CBAS will not become an efficient method to discover biological findings. CBAS takes a role to assure conclusions derived by hypothesis-driven approaches, nothing more, nothing less.

In addition to what we describe in the above response about the ability of CBAS to identify novel findings, the reviewer is absolutely correct that CBAS also provides a valuable way of richly testing hypotheses. Having a general method to falsify hypotheses is extremely important for evaluating interpretations of complex data. Otherwise, as we describe in the manuscript, hypothesis-driven analyses suffer from confirmation bias.

2. Hypothesis exists in the definition of CBAS language.
In genome-wide analyses, the language for analyses is determined. In contrast, there are various definitions of the language for behavioral sequences.

To be fair, even in genome-wide analyses decisions must be made as to what is compared. For GWAS, only SNPs are compared between groups, for WES, only protein coding regions are compared, and even with WGS there is a comparison to a reference genome. We have added an extra sentence to the discussion section to highlight further the relationship between choice of language and bias and the critical need to keep that in mind while interpreting CBAS. "It is critical to remember and consider the biases imposed by the language when interpreting the results of CBAS."

In the two-step task, this study ignored the choice of the second step, c2. This is one hypothesis. The standard stay probability analyses ignore the second choices, but the fitting of hybrid models is affected by the second choices. The definition of CBAS language restricted the feature of behavioral sequences. The extension of sequence length from 3 to 4 is meaningless, because state transitions are independent by trials. The present definition of CBAS language restricted the analysis in the hypothesis common to the standard stay probability analysis. If we attempt to examine longer sequences, then one effective definition is to describe trial-type sequences, each of which is one of 8 trial types (choice1/choice2) x (common/rare) x (rewarded/unrewarded).

The reviewer points to a different language that could have been chosen as the basis of the CBAS applied to the two-step task. This is something that we considered but ultimately chose the language we presented for its simplicity and its potential (borne out by the ultimate finding) to identify previously unknown information about this task and its relationship to CBIT. We also included a discussion about the decision to combine the two choices at the second stage of the task into a single token. It introduces a bias as to what CBAS can identify, but we deemed that to be a worthwhile trade-off given the possibility for data-limitations (the same reason we chose to evaluate sequences up to 4 long for this task).

Anyway, the definition of CBAS language contains hypotheses. Therefore, CBAS is not truly data-driven approach.

We appreciate this perspective. In our view, data-driven and hypothesis-driven analyses lie along a spectrum, with a definition that is not black and white. Data-driven analyses impose less bias; however, biases still exist. We intentionally did not call CBAS an unbiased analysis and worked to make its biases explicit. However, we realize that in our introduction we fell into the trap of categorizing data-driven and hypothesis-driven approaches as two types of analyses and did not portray them as lying along a spectrum. We have updated the text of the introduction to capture better the true spectrum of these approaches. "Approaches for analyzing such data range from top-down/model-dependent on one end of a spectrum to bottom-up/data-driven on the other end of the spectrum[17,18]. Methods that tend towards the model-dependent end of the spectrum are substantially more prevalent in the analysis of behavior; however, methods that tend toward the data-driven end of the spectrum dominate in other areas with rich and complex findings and afford the possibility of addressing some of the many limitations of top-down approaches."

Minor comments:
L480 eq.(4): In general, the variance should be divided by n– 1 instead of n for unbiased estimation. Please confirm whether division by n is correct in Romano-Wolf scheme.

From a statistical point of view, unbiasedness is actually not a good reason to divide by n-1 rather than n. In fact, dividing by n results in a smaller mean squared error because it has a smaller variance. There is no consensus in statistics and among statistical software which is preferable. In fact, dividing by n+1 leads to a variance estimate with even smaller mean squared error if the underlying distribution is bivariate normal.

However, and more importantly, the scale of any constant factor plays no role in carrying out the permutation test. This is because if you multiply the test statistic by n, n-1, or anything else, all the recomputed test statistics based on permuted values are multiplied the same way as well. This is true for bootstrap as well. Only if we were to be using a parametric method based on, for instance, a Gaussian approximation (which we do not here), would it matter.

L579-580: Description $Q_t^{MB} = max\ (Q_t^{stage2}(s, c_2))$ is wrong. Q_t^{MB} is a function of c_1, $Q_t^{MB}(c_1)$, and determined with choice-dependent transition probabilities. Correct the definition of Q_t^{MB}.

Thank you for catching this. We have corrected the equation.

L581-582: Description includes many mistakes about commas and subscriptions. I guess that you intended to describe as $Q_{t+1}^{MF_0}(c_{1,t}) = (1-\alpha)Q_t^{MF_0}(c_{1,t}) + Q_t^{stage2}(s_t, c_{2,t})$ and $Q_{t+1}^{MF_1}(c_{1,t}) = (1-\alpha)Q_t^{MF_1}(c_{1,t}) + r_t$.

Thank you for catching this also. We have corrected the equations.

L262, L702, L706: Italic x generally indicates a variable in mathematical forms. If you intend to represent the symbol of "times" (×), use the roman font "x" or the symbol of "times" (×) by mathematical editing tools.

Thank you for catching this. We have corrected these instances.

Reviewer #3 (Remarks to the Author):
This study introduces the Choice-Wide Behavioral Association Study (CBAS), a novel data-driven method for systematically identifying behavioral components from sequences of discrete choices. Inspired by the logic of genome-wide association studies (GWAS), CBAS evaluates the occurrence of all discrete choice sequences up to a user-defined length and uses a resampling-based multiple comparisons correction (using Romano-Wolf method) to identify sequences that either significantly differ between groups or

correlate with a covariate of interest. The authors apply CBAS to three example tasks across three species, namely spontaneous alternation in flies, the two-step task in humans, and spatial alternation in rats, demonstrating the breadth of the method. In each case, CBAS identifies interpretable behavioral differences that are either inconsistent with or go beyond the original conclusions of prior model-dependent analyses and the work that the data came from. For instance, for the rat spatial alternation task, CBAS reveals that the hippocampus supports a specific exploratory strategy characterized by directional inertia and short-range arm transitions, reframing the hippocampal contribution away from simple memory allocation.

The paper addresses an important gap in behavioral neuroscience methodology, and the three diverse applications make a compelling case for the generality of the approach. The statistical framework is rigorous, and the results are clearly presented.

Thank you very much for this positive assessment and your helpful comments.

Our comments primarily focus on clarifying key methodological choices and strengthening the interpretation of the results.

Major Comments:
1. The GWAS analogy would benefit from a more explicit treatment of the conceptual equivalences (e.g. line 85). The authors motivate CBAS by drawing on GWAS, WES, and WGS, which is a compelling framing. However, the analogy is stated at a high level without spelling out the specific parallels. For instance, it would help the reader to understand how a choice sequence maps onto a base pair or a genetic variant in more detail, what the behavioral equivalent of a phenotype is in this framework, and how the resampling-based correction serves the same role as genome-wide significance thresholds. Making these correspondences explicit would strengthen the conceptual contribution of the paper and help readers from different backgrounds appreciate the logic of the approach.

Thank you for this excellent suggestion. We have added text in the manuscript fleshing out the analogy between the genomic methods and CBAS as you suggest: "We draw upon the analogy with the genomic methods (GWAS/WES/WGS) to explain the function and goals of CBAS. Genomic methods aim to identify variation in the genome (at the level of DNA base pairs or larger structural changes) that is associated with a phenotype of interest, such as disease. These variants can include single nucleotide polymorphisms (SNPs), insertions/deletions, or structural changes, and may occur in coding or noncoding regions. The nature of the variants identified guides understanding of the biological mechanisms underlying the phenotype or disease. In these terms, CBAS aims to identify variation in behavioral structure (at the level of sequences of discretized units such as choice) that is associated with a phenotype of interest such as a strain of fly or an index of compulsivity revealed by answers to questionnaires in humans. The structure of the sequences of choices guides understanding into the behavioral algorithms underlying the phenotype."

2. The inability of CBAS to operate on a single group without a covariate is a notable limitation that warrants further discussion. The authors briefly acknowledge in the Discussion that CBAS currently requires either a group comparison or a covariate of interest and cannot evaluate a single group in an unsupervised manner. However, this seems like a meaningful constraint on the method's applicability, particularly in exploratory settings where no clear comparison group or covariate exists. It would be helpful if the authors expanded on this limitation, discussed whether there are principled extensions that could address it, and clarified whether existing unsupervised approaches (e.g., clustering-based methods) could be combined with CBAS in such cases.

We have added further text to the discussion section to describe possibilities for surmounting this limitations: "If there is only a single group of subjects with no experimentally defined covariate, but a regular clustering algorithm detects structure, then CBAS can be run on the output of that. In future work, we will examine an unsupervised application of CBAS in which robust cluster structure arises from random initial conditions."

3. The generalizability of CBAS beyond discrete choices is not fully addressed. CBAS is built around the discretization of behavior into choices, which works naturally for the three tasks presented. However, many behavioral paradigms do not yield such clean discrete representations and many research approaches are using more continuous paradigms. The authors briefly note this limitation but do not discuss what strategies might be used to extend CBAS to richer or higher-dimensional behavioral spaces, for instance through unsupervised discretization of continuous trajectories. A more thorough exploration of this boundary condition would help the reader understand where CBAS is and is not applicable.

We have added further text to the discussion to expand on the continuous vs discretized behavior separation and the limitations of CBAS therein: "For some continuous data, this limitation can be overcome by imposing discretization or tokenization of the data. For example, if the behavior measures the continuous movement of an animal in space, as in an elevated plus maze[63] , then one can discretize the location of the animal in units of time, or a string of discretized locations independent of the time spent in those locations. As another example, for acoustic startle and prepulse inhibition[64] the continuous startle measurement could be discretized into a range of startle magnitudes. For other types of continuous data, such artificial discretization might not be ideal. What CBAS then requires is a metric to determine when two continuous behavioral trajectories are sufficiently close to be counted as the same. In future work, it will be interesting to explore methods for creating and validating such metrics."

4. The sensitivity of the conclusions to the choice of maximum sequence length is not assessed. The maximum sequence length is a key user-defined parameter in CBAS, set to 10 for flies, 4 for humans, and 6 for rats, but the rationale for these specific choices is not clearly explained, and no sensitivity analysis is provided. It is not obvious that the key conclusions would hold under different choices of this sequence length parameter. For example, would the same interpretable patterns in the fly data emerge if sequences up to length 6 or 8 were used instead of 10? The authors should either provide a justification for these choices or demonstrate that the results are robust to reasonable variation in this parameter.

Thank you for this important suggestion. We have adding clarifying text to explain our decision for each of the species analyzed. Flies: "The choice of 10 was made to allow for the possibility to identify rich structure that differentiates the groups." Humans: "We chose to limit CBAS to a maximum of only 4 choices long as that length of sequence captures the critical transition for this behavior between a reward at the end of the prior trial and the choice at the first stage of the next trial." Rats: "We opted for sequences with a maximum of 6 choices long because sequences that long can contain all of the ways that rewards can be received for this task and provide rich structure for the many ways in which reward can be missed."

Furthermore, as we did with figure 3, we took advantage of the large number of subjects in the fly dataset to explore different maximum sequence lengths. We have added text and a supplemental figure showing that the conclusion that we find with using sequences up to 10 choices long also hold if we evaluated the dataset using sequences up to 5 choices long. When using sequences up to 3 or 2 long we are still able to show that the data are inconsistent with the differences in the lines being due to a different in turn bias, however, we are no longer able to distinguish between the persistence rate and distribution. "We additionally took advantage of the large group size to evaluate the sensitivity of the conclusions to the choice of maximum sequence length evaluated by CBAS. Using only a maximum sequence length of 5 choices supports the same conclusion found with using a maximum sequence length of 10 choices (Fig S2a). However, with a maximum sequence length of 3 or 2 choices it is no longer possible to separate

between the persistence rate and persistence distribution models, even though such short sequence lengths still rule out the turn bias model (Fig S2b)."

5. The distinction between 'persistence rate' and 'persistence distribution' is not clearly explained (e.g. line 156). The authors compare three generative models of fly behavior, namely turn bias, persistence rate, and persistence distribution, and show that only the persistence distribution model matches the CBAS output. However, the difference between persistence rate and persistence distribution is not made sufficiently clear. Specifically, it is not obvious what additional information the distribution captures beyond the rate, and why this distinction matters for generating realistic sequences and why this distinction is functionally useful.

Apologies for the lack of clarity. We have added text clarifying the difference between persistence rate and persistence distribution, as well as the implication of the fact that the persistence distribution was a better fit for the fly behavior. "The virtual twins generated with the persistence distribution of the flies differ from the virtual twins generated with the persistence rate of the flies by utilizing the full distribution of their tendency to continue in the same direction for each possible number of turns instead of just the average persistence rate of each animal. This means using the full distribution instead of just the central tendency of this distribution. The better fit of the virtual twins using the full persistence distribution indicates that there is meaningful information for the behavior of the flies beyond just their central tendency to persist in turning in the same direction."

6. It is unclear how the replication dataset addresses the circularity concern (as in Figure 6). The authors use the replication dataset to validate the hypothesis of reduced directional inertia in hippocampal lesion rats. However, the replication uses the same task, the same apparatus, and the same general experimental design as the initial dataset and is simply a smaller independent cohort. Given that the hypothesis was derived directly from the CBAS output on the initial dataset, and that the same metric (transition normalized directional inertia) is applied in the replication, it is not clear that this fully resolves the circularity concern the authors themselves raise. Could the authors clarify what specific aspect of circularity the replication is intended to address, and what would constitute a stronger test?

Sorry for being unclear. The circularity we were worried about is that we had used the sequences that CBAS had identified as being discriminative on the original cohort to create a hypothesis about a measure (transition normalized directional inertia) that would be distinctive. To ensure that we had not over-fit to the original cohort, we sought to test whether the measure applied in a new and independent cohort of animals. We have added text clarifying that the animals in replication dataset were not involved in the initial analysis. "Importantly, the data from these new lesion and control rats were neither a part of the initial CBAS evaluation nor the comparison of the groups via the transition normalized directional inertia metric."

7. The notion of 'model-inconsistent' choices needs to be better grounded in decision theory. The authors identify sequences of the form 1A2 and 2B1, where a subject is rewarded on the common side but then selects the image that will rarely return them there, as 'model-inconsistent.' While this is an interesting empirical observation, the conceptual status of these choices is not clearly defined. They are inconsistent with both model-based and model-free learning, but what alternative decision process would produce them? The authors suggest these choices may be related to CBIT without explaining the mechanism. This example may provide a test case how to interpret the CBAS-based discovery for functional implication with respect to disorders or other mechanisms in the brain. A more careful discussion of what model-inconsistent choices reflect, whether they constitute a distinct behavioral mode, a failure of attention, or something else entirely, would substantially strengthen the interpretation of the two-step task results.

This is a good point. In the discussion section, we have now added two potential hypotheses for how model-inconsistent choice could show positive correlation with CBIT. "Possible ways in which model-

inconsistent choices positively correlate with CBIT include an increase in exploration in the context of the lower task success that has been shown to associate with OCD[53]. That lower task success could lead to failures in both model-based and model-free decision making, which then manifests as more model inconsistent choices. Another route would be an increase in a desire for symmetry that can be a large component of OCD[54]. That desire for symmetry could lead to an evening out of choices to both of the first images independent of the potential for success of those choices. These would then naturally be model inconsistent."

Minor Comments:

1. Figure 1b: The rate values reported (e.g., 9.9 per animal for the CA strain) lack a unit. It would be helpful to clarify whether this is sequences per 250 choices, per session, or some other normalization.

The rate is the number of choices per individual subject during the choice used for the criterion. We have added this clarification to the figure legends.

2. Line 795: typo: "odel" should be "model."

Thank you for finding that typo. We have corrected it.

3. Line 362: The section header reads "DISCUSSSION" and should be corrected to "DISCUSSION."

Thank you for finding that typo. We have corrected it.

Reviewer #4 (Remarks to the Author):
I co-reviewed this manuscript with one of the reviewers who provided the listed reports. This is part of the Nature Communications initiative to facilitate training in peer review and to provide appropriate recognition for Early Career Researchers who co-review manuscripts.

Thank you.
