# Supplementary material for: The choice-wide behavioral association study: data-driven identification of interpretable behavioral components
Source: Nat Commun. 2026 Aug 13;17:9746. doi: 10.1038/s41467-026-76681-3 (PMC13572513; doi:10.1038/s41467-026-76681-3)
Supplement: Supplementary file 3 [file 41467_2026_76681_MOESM3_ESM.pdf]

nature portfolio

Corresponding author(s): David Kastner

Last updated by author(s): Jul 11, 2026

Reporting Summary

Nature Portfolio wishes to improve the reproducibility of the work that we publish. This form provides structure for consistency and transparency in reporting. For further information on Nature Portfolio policies, see our Editorial Policies and the Editorial Policy Checklist.

Statistics

For all statistical analyses, confirm that the following items are present in the figure legend, table legend, main text, or Methods section.

n/a	Confirmed	
☐	☒	The exact sample size (n) for each experimental group/condition, given as a discrete number and unit of measurement
☐	☒	A statement on whether measurements were taken from distinct samples or whether the same sample was measured repeatedly
☐	☒	The statistical test(s) used AND whether they are one- or two-sided<br>*Only common tests should be described solely by name; describe more complex techniques in the Methods section.*
☒	☐	A description of all covariates tested
☐	☒	A description of any assumptions or corrections, such as tests of normality and adjustment for multiple comparisons
☐	☒	A full description of the statistical parameters including central tendency (e.g. means) or other basic estimates (e.g. regression coefficient) AND variation (e.g. standard deviation) or associated estimates of uncertainty (e.g. confidence intervals)
☐	☒	For null hypothesis testing, the test statistic (e.g. F, t, r) with confidence intervals, effect sizes, degrees of freedom and P value noted<br>*Give P values as exact values whenever suitable.*
☒	☐	For Bayesian analysis, information on the choice of priors and Markov chain Monte Carlo settings
☒	☐	For hierarchical and complex designs, identification of the appropriate level for tests and full reporting of outcomes
☒	☐	Estimates of effect sizes (e.g. Cohen's d, Pearson's r), indicating how they were calculated

Our web collection on statistics for biologists contains articles on many of the points above.

Software and code

Policy information about availability of computer code

Data collection	For the data generated for the study, data was collected using Spike Gadget Trodes software.
Data analysis	Data was analyzed using custom designed software within the IGOR Pro (version 8.0) by Wavemetrics program. Code is posted at: https://github.com/dbkastner/CBAS.

For manuscripts utilizing custom algorithms or software that are central to the research but not yet described in published literature, software must be made available to editors and reviewers. We strongly encourage code deposition in a community repository (e.g. GitHub). See the Nature Portfolio guidelines for submitting code & software for further information.

Data

Policy information about availability of data

All manuscripts must include a data availability statement. This statement should provide the following information, where applicable:

- Accession codes, unique identifiers, or web links for publicly available datasets
- A description of any restrictions on data availability
- For clinical datasets or third party data, please ensure that the statement adheres to our policy

Data generated for this study is available at: https://github.com/dbkastner/CBAS.

Research involving human participants, their data, or biological material

Policy information about studies with human participants or human data. See also policy information about sex, gender (identity/presentation), and sexual orientation and race, ethnicity and racism.

Reporting on sex and gender	Human dataset used in this study is described in the original publication: https://elifesciences.org/articles/11305.
Reporting on race, ethnicity, or other socially relevant groupings	Human dataset used in this study is described in the original publication: https://elifesciences.org/articles/11305.
Population characteristics	Human dataset used in this study is described in the original publication: https://elifesciences.org/articles/11305.
Recruitment	Human dataset used in this study is described in the original publication: https://elifesciences.org/articles/11305.
Ethics oversight	Human dataset used in this study is described in the original publication: https://elifesciences.org/articles/11305.

Note that full information on the approval of the study protocol must also be provided in the manuscript.

Field-specific reporting

Please select the one below that is the best fit for your research. If you are not sure, read the appropriate sections before making your selection.

[x] Life sciences [ ] Behavioural & social sciences [ ] Ecological, evolutionary & environmental sciences

For a reference copy of the document with all sections, see nature.com/documents/nr-reporting-summary-flat.pdf

Life sciences study design

All studies must disclose on these points even when the disclosure is negative.

Sample size	Sample size was evaluated after the fact with power calculations using CBAS.
Data exclusions	Data exclusions are described in the manuscript. Briefly, no human or rat subjects were excluded. Flies that did not do at least 250 turns were excluded from the analysis.
Replication	The rat result was replicated and shown in figure 6 of the manuscript. This was the only results for which replication was attempted. Otherwise reproducibility was prioritized through the rigorous statistical analyses applied within CBAS.
Randomization	For the human and fly datasets no randomization was used. For the rat dataset, rats were randomly assigned to either lesion or control surgery. For the rat dataset, there were no covariates analytically control for as litter mates were used and individual from both sex were used. For the fly dataset there were no covariates listed in the public repository to control for. And for the human dataset all subjects were used with on their own for correlation with CBAS.
Blinding	For the rat dataset, experimenters were blinded to the lesion status of the rat during collection of the data and evaluation of the lesion via CT. For the fly an human datasets descriptions are in the original papers describing the work. All analyses were done independent of groups status.

Reporting for specific materials, systems and methods

We require information from authors about some types of materials, experimental systems and methods used in many studies. Here, indicate whether each material, system or method listed is relevant to your study. If you are not sure if a list item applies to your research, read the appropriate section before selecting a response.

Materials & experimental systems

n/a	Involved in the study	
x		Antibodies
x		Eukaryotic cell lines
x		Palaeontology and archaeology
	x	Animals and other organisms
x		Clinical data
x		Dual use research of concern
x		Plants

Methods

n/a	Involved in the study	
x		ChIP-seq
x		Flow cytometry
x		MRI-based neuroimaging

Animals and other research organisms

Policy information about studies involving animals; ARRIVE guidelines recommended for reporting animal research, and Sex and Gender in Research

Laboratory animals	Long Evans rats ~4-6 months old were used for the study. Outbred, long Evans rats were used. Rats were bred in house with founder animals purchased from Charles Rivers and acquired from the Medical College of Wisconsin.
Wild animals	NA
Reporting on sex	Males and females in all species (humans, rats, and flies) were used and combined into single groups for analysis.
Field-collected samples	NA
Ethics oversight	For the rat data, the UCSF Institutional Animal Care and Use Committee approved the data collection.

Note that full information on the approval of the study protocol must also be provided in the manuscript.

Plants

Seed stocks	NA
Novel plant genotypes	NA
Authentication	NA
